# Development of End-to-End Artificial Intelligence Models for Surgical Planning in Transforaminal Lumbar Interbody Fusion

**DOI:** 10.3390/bioengineering11020164

**Published:** 2024-02-08

**Authors:** Anh Tuan Bui, Hieu Le, Tung Thanh Hoang, Giam Minh Trinh, Hao-Chiang Shao, Pei-I Tsai, Kuan-Jen Chen, Kevin Li-Chun Hsieh, E-Wen Huang, Ching-Chi Hsu, Mathew Mathew, Ching-Yu Lee, Po-Yao Wang, Tsung-Jen Huang, Meng-Huang Wu

**Affiliations:** 1 International Ph.D. Program in Medicine, College of Medicine, Taipei Medical University, Taipei 11031, Taiwan; 2 Department of Spine Surgery, Military Hospital 103, Vietnam Military Medical University, Hanoi 100000, Vietnam; 3 School of Computer and Communication Sciences, Swiss Federal Institute of Technology in Lausanne, 1015 Lausanne, Switzerland; hle@cs.stonybrook.edu; 4 Department of Trauma-Orthopedics, College of Medicine, Pham Ngoc Thach Medical University, Ho Chi Minh City 700000, Vietnam; giamtm@pnt.edu.vn; 5 Department of Pediatric Orthopedics, Hospital for Traumatology and Orthopedics, Ho Chi Minh City 700000, Vietnam; 6 Institute of Data Science and Information Computing, National Chung Hsing University, Taichung City 402, Taiwan; 7 Biomedical Technology and Device Research Laboratories, Industrial Technology Research Institute, Hsinchu 31057, Taiwan; 8 Department of Radiology, School of Medicine, College of Medicine, Taipei Medical University, Taipei 11031, Taiwan; 9 Department of Medical Imaging, Taipei Medical University Hospital, Taipei 11031, Taiwan; 10 Research Center of Translational Imaging, Taipei Medical University Hospital, Taipei 11031, Taiwan; 11 Department of Materials Science and Engineering, National Yang Ming Chiao Tung University, Hsinchu 30013, Taiwan; 12 Department of Mechanical Engineering, National Taiwan University of Science and Technology, Taipei 106, Taiwan; 13 Department of Biomedical Engineering, Colleges of Engineering and Medicine, University of Illinois Chicago, Chicago, IL 60607, USA; 14 Department of Orthopedics, Taipei Medical University Hospital, Taipei 11031, Taiwan221002@h.tmu.edu.tw (P.-Y.W.);; 15 Department of Orthopedics, School of Medicine, College of Medicine, Taipei Medical University, Taipei 11031, Taiwan; 16 TMU Biodesign Center, Taipei Medical University, Taipei 11031, Taiwan

**Keywords:** spinal fusion, interbody cage, sagittal balance, artificial intelligence, machine learning, spinal parameters

## Abstract

Transforaminal lumbar interbody fusion (TLIF) is a commonly used technique for treating lumbar degenerative diseases. In this study, we developed a fully computer-supported pipeline to predict both the cage height and the degree of lumbar lordosis subtraction from the pelvic incidence (PI-LL) after TLIF surgery, utilizing preoperative X-ray images. The automated pipeline comprised two primary stages. First, the pretrained BiLuNet deep learning model was employed to extract essential features from X-ray images. Subsequently, five machine learning algorithms were trained using a five-fold cross-validation technique on a dataset of 311 patients to identify the optimal models to predict interbody cage height and postoperative PI-LL. LASSO regression and support vector regression demonstrated superior performance in predicting interbody cage height and postoperative PI-LL, respectively. For cage height prediction, the root mean square error (RMSE) was calculated as 1.01, and the model achieved the highest accuracy at a height of 12 mm, with exact prediction achieved in 54.43% (43/79) of cases. In most of the remaining cases, the prediction error of the model was within 1 mm. Additionally, the model demonstrated satisfactory performance in predicting PI-LL, with an RMSE of 5.19 and an accuracy of 0.81 for PI-LL stratification. In conclusion, our results indicate that machine learning models can reliably predict interbody cage height and postoperative PI-LL.

## 1. Introduction

Over the past few decades, transforaminal lumbar interbody fusion (TLIF) has been commonly used to treat lumbar degenerative diseases, demonstrating the benefits of achieving satisfactory arthrodesis through a unilateral approach with minimal impingement on neural components [1,2]. In addition to relieving spinal nerve compression, the primary objective of TLIF is to restore sagittal balance and the intervertebral body height [3,4,5].

In terms of sagittal alignment, several studies have reported a close relationship between postoperative sagittal malalignment and postoperative residual symptoms in patients with lumbar fusion [5,6]. Among the parameters of spinal alignment, subtraction of lumbar lordosis (LL) from the pelvic incidence (PI) is a crucial indicator of postoperative outcomes after short-segment lumbar interbody fusion for lumbar pathologies. Patients with PI-LL (PI minus LL) mismatch have increased risks of adjacent segment disease (ASD), late surgical complications, and revision surgery [7,8,9]. Therefore, postoperative alignment prognosis, especially for critical parameters such as PI-LL, is required for optimal preoperative planning for lumbar fusion. However, predicting postoperative alignment in patients is challenging. Ailon et al. [10] reported that only 42% of cases were accurately predicted by 17 experienced surgeons specializing in treating spinal deformity. Although various methods exist for predicting postoperative parameters in patients with adult spinal deformity [11,12], a method for predicting the value of PI-LL in TLIF procedures still needs to be developed.

Selecting an interbody cage with the correct height is a crucial aspect of lumbar interbody fusion. Utilizing an undersized cage may result in the inability to restore the intervertebral height and segmental lordosis, as well as in complications such as pseudarthrosis and cage migration [13,14,15]. By contrast, utilizing an oversized cage may increase the likelihood of nerve root compression, ASD, or cage subsidence [15]. In clinical practice, the cage height has long been selected subjectively by surgeons depending on their operational experience. Few studies have predicted the height of fusion cages on the basis of the intervertebral height of the pathological segment [16] or the anterior and posterior disc height on a preoperative computed tomography (CT) image [17]. However, in severe degenerative diseases, such as spondylolisthesis and spinal deformity, when the disc height is greatly reduced, these methods are often inaccurate. Thus, estimating the height of interbody cages remains a challenge.

The choice of the cage height affects sagittal balance (and vice versa), and preoperative spinal parameters play a key role in determining the appropriate size of the implanted device for achieving favorable parameters after surgery [16,18]. Therefore, it is imperative to develop regression models for predicting interbody cage height and postoperative parameters based on preoperative data. However, manual measurements are time-consuming for obtaining all parameters and are prone to rater-dependent errors. Presently, automated tools involving artificial intelligence (AI) are employed to enhance the accuracy and efficiency of measuring spinal alignment parameters from radiographic images [12,19]. Despite these advancements, there is a notable gap in the literature as, to the best of our knowledge, the integration of AI-derived parameters into regression models for surgical planning remains underdeveloped. Moreover, while AI has found broad application across various surgical domains, its utilization in TLIF surgery has predominantly been observed in predicting postoperative clinical outcomes, with limited clarity in its integration into surgical planning [20,21]. This study aims to develop a dedicated pipeline utilizing AI and machine learning (ML) to reliably predict interbody cage height and postoperative PI-LL in TLIF surgery based on preoperative X-ray images.

## 2. Materials and Methods

### 2.1. Patient Selection

A total of 311 patients who underwent L4–L5 TLIF surgery between January 2019 and December 2021 at our institution were included in this retrospective study. The following patients were included: (1) patients with lumbar degenerative diseases, such as lumbar disc herniation, lumbar spinal stenosis, and spondylolisthesis; (2) patients who underwent TLIF surgery to implant a single interbody cage; and (3) patients who did not experience any complications, such as cage migration, pseudarthrosis, or fusion failure, and did not require revision surgery because of cage problems or ASD during the follow-up period (at least 6 months). We chose a 6-month follow-up period to capture immediate postoperative outcomes and identify potential complications within the early recovery phase, aligning with common practices in spine surgery research.

The following patients were excluded: (1) patients with a history of lumbar fractures or patients who received a diagnosis of one-segment lumbar degenerative disease at other levels, multiple lumbar degenerative diseases, lumbar scoliosis, spinal tumors, or severe osteoporosis; (2) patients who received two interbody cage implants; (3) patients with unstandardized sagittal radiographs with low image quality for segmentation or radiographs lacking a femoral head; and (4) patients who experienced neurological or neuromuscular episodes during the follow-up period, as they could have unsatisfactory postoperative outcomes, leading to errors in training the model.

In addition to preoperative and postoperative X-ray images and the size of the surgically implanted interbody fusion cage, the demographics of each patient were obtained. Standing lateral X-ray images in a neutral position were chosen due to their superior quality and standardization compared to intraoperative X-ray images. Furthermore, variations in spinal parameters across different postures may introduce inconsistencies among patients [22,23]. Consequently, to minimize segmentation bias and errors in parameter measurements, only one lateral neutral radiograph was selected for each patient. Imaging data were obtained using a Radnext 50 X-ray machine from Hitachi Global (Tokyo, Japan). X-ray exposure parameters were set to 78 kVp and 60–100 mAs, with all machines equipped with an Automatic Exposure Control system. A certified radiographer conducted an examination for each image to ensure the visibility of vertebrae from T12 to the lower sacrum, two femoral heads, open intervertebral disc spaces, visible spinous processes, and the superimposed posterior margins of each vertebral body. The Exposure Index value was additionally employed as part of our quality control measures.

### 2.2. X-ray Segmentation and Feature Extraction

**Figure 1:**
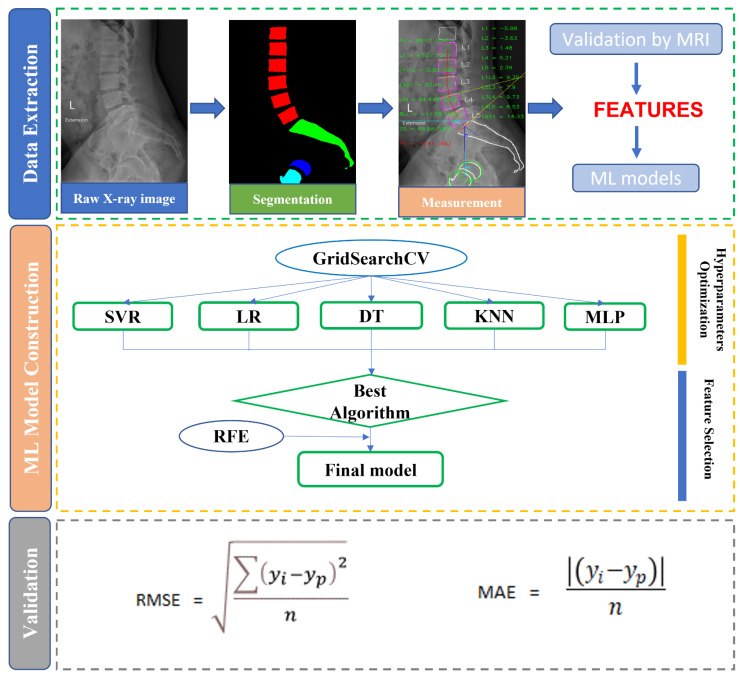
Study flowchart depicting four subprocesses: data cohort collection, feature extraction, feature validation, and ML model construction and validation. ML: machine learning; SVR: support vector regression; LR: LASSO regression; DT: decision tree; KNN: *K*-nearest neighbor; MLP: multilayer perceptron; RFE: recursive feature elimination; RMSE: root mean square error; MAE: mean absolute error.

A pretrained BiLuNet model was employed to segment each input X-ray image into various semantic regions, including the L1, L2, L3, L4, and L5 regions; a sacrum region; and two femoral head regions (Figure 1) [24]. The model demonstrated proficient performance in lumbar spine segmentation, as substantiated by the results presented in our previous study [25]. After resizing the original image to 512 × 512 pixels, the model generated an output image with four labels: background, lumbar vertebral regions, sacrum, and two femoral heads. Nearest-neighbor interpolation was then used to resize the segmented image to its original size. Based on the contours of the segmented areas, a computer vision algorithm obtained multiple corner points to measure the spinal parameters on preoperative X-ray images. This process employed OpenCV tools to calculate contours, fit appropriate polygons, determine the corner points of the polygons, and measure the spinal parameters. Subsequently, these features were combined with four demographic features—namely, age, gender, body height, and fusion indication—to derive input features for ML algorithms. These factors were explored in previous studies [16,26], demonstrating their impact on spinal parameters and TLIF surgery outcomes. Consequently, we aimed to integrate these clinical factors with image features to enhance the overall predictive capability of the model. Finally, the PI-LL value was measured from the postoperative X-ray image by two experienced surgeons (C.-Y.L. and M.-H.W.) and served as a validation standard for ML models.

To assess the measurement precision of the BiLuNet model, two authors (A.T.B. and G.M.T.) independently measured the aforementioned parameters using magnetic resonance imaging (MRI) and compared their results with those of the model. Since the MRI angle parameters in the supine position differ from those obtained from standing X-ray images, only bone distance features were selected to evaluate interobserver reliability.

### 2.3. ML Implementation

We divided our ML pipeline into three steps: data extraction, model building, and validation (Figure 1). All steps were performed using Python 3.7 and scikit-learn 1.1.2 package [27].

#### 2.3.1. Data Preprocessing

The categorical features were encoded as one-hot embeddings, and no normalization was applied to them. Each missing value of continuous variables was examined and replaced by the mean value of each parameter. Due to distinct units and large differences between feature ranges, the *z*-score was employed in the data normalization step [28]. This involved subtracting the mean and dividing by the standard deviation for each feature. Z-score normalization was chosen for its ability to standardize scales, accommodating diverse units and magnitudes within the dataset while preserving distribution characteristics. This widely accepted practice enhances result comparability and interpretability.

#### 2.3.2. Regression Models

Various ML models were evaluated to determine their performance for the aforementioned features. These models included five regression algorithms: decision tree (DT), LASSO regression (LR), support vector regression (SVR), *K*-nearest neighbor (KNN), and multilayer perceptron (MLP). Hyperparameter optimization was conducted for each ML algorithm through the GridSearchCV method to achieve improved results. The algorithm with the highest performance was selected as the baseline model to construct the final ML model. After baseline ML models were obtained for either cage height or postoperative PI-LL prediction, we employed Recursive Feature Elimination (RFE) for feature selection. RFE operates iteratively, removing the least crucial features and rebuilding the model with the remaining features.

To determine the optimal number of features, an RFE loop was performed with cross-validation (RFECV function). The mean absolute error (MAE) of the model was then calculated across all repetitions and folds of the RFECV function. Generally, the scikit-learn library represents the MAE as a negative value to maximize it. Therefore, a model with a large negative MAE value is regarded as superior for RFE visualization. After the RFE process, the final model was built using the optimal subset of features, with the SHapley Additive exPlanations (SHAP) value indicating the importance of each feature in model prediction [29]. In particular, the computation of SHAP values involves the iterative comparison of a model’s predictions with and without the inclusion of a specific feature. This process is carried out for each feature and every sample in the dataset.

### 2.4. Statistical Analysis and Measurement Metrics

A five-fold cross-validation (*k* = 5) was performed to assess the efficacy of the ML regression algorithms. The model was then trained on *k −* 1 data splits, and the trained model was tested on the remaining held-out split. Subsequently, the performance of each model was averaged across all data splits for comparison. This cross-validation scheme provided a more reliable test result than that derived using a single fixed testing data split, especially when training data were limited. It also guaranteed that each data point was tested exactly once. Furthermore, we repeated this process five times with a different random data split and reported the mean and variance.

To compare the performance of all ML algorithms, both the root mean square error (RMSE) and the MAE of each model were calculated. The testing error in each case was then visualized to evaluate the accuracy of prediction. To examine the reliability of features in the deep learning model, the intraclass correlation coefficient (ICC) was calculated using SPSS version 18.0 (SPSS, Chicago, IL, USA). The 95% confidence interval of the ICC estimate suggests poor reliability for values below 0.5, moderate reliability for values between 0.5 and 0.75, adequate reliability for values between 0.75 and 0.9, and excellent reliability for values greater than 0.9 [30]. Schwab classification [31] was then performed with three levels of PI-LL, and the final model was evaluated in terms of its ability to stratify postoperative PI-LL based on the accuracy index and F1-score. This classification system was chosen due to its widespread acceptance and relevance in clinical practice within the spinal surgery community [32,33]. Generally, a PI-LL value below 10° yields a modifier of 0, a value between 10° and 20° yields a modifier of 1, and a value greater than 20° yields a modifier of 2 [31].

## 3. Results

### 3.1. Patient Characteristics

**Figure 2:**
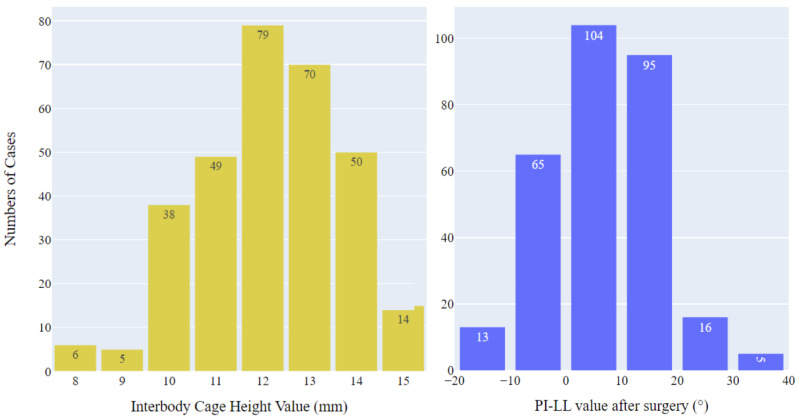
Distribution of actual interbody cage heights and postoperative PI-LL values.

This study included 126 men and 185 women, with a mean age of 64.08 years (standard deviation: 11.19) and a mean body height of 159.45 cm (standard deviation: 8.39). In total, 88 patients had lumbar disc herniation, 154 patients had lumbar spinal stenosis, and 69 patients had lumbar spondylolisthesis. Figure 2 depicts the ground truth distribution of two predictable parameters. Most of the cases (149/311 cases) had cage heights of 12–13 mm, with only few cases having fusion cage heights of 8, 9, and 15 mm. Similar uneven distribution was observed in PI-LL values after surgery, with the majority of patients having PI-LL values ranging from 0 to 20. These unbalanced proportions posed a challenge for the optimization of the ML algorithms.

### 3.2. Performance of ML Algorithms

A total of 53 features were extracted from preoperative X-ray images using a deep learning model (Supplementary Table S1). These features demonstrated highly reliability, as evidenced by interobserver reliability within an ICC range of 0.78–0.947 (Supplementary Table S2). These results affirm the robust performance of the deep learning model in accurately measuring spinal parameters. Following the inclusion of 4 clinical features, a total of 57 features were input into the regression models.

Subsequent experiments were conducted to determine the optimal parameters of each ML algorithm in predicting both cage height and postoperative PI-LL. Table 1 enumerates the ranges of all scrutinized hyperparameters and their corresponding optimal values. Upon comparison of the five algorithms with optimal parameters, LR exhibited superior performance in predicting the cage height, with an RMSE of 1.06 and an MAE of 0.76. Notably, SVR emerged as the optimal model for predicting postoperative PI-LL, displaying the lowest RMSE (5.4) and MAE (4.15) among the algorithms considered, followed by LR, MLP, KNN, and DT (Table 2). Consequently, LR was selected as the baseline model for predicting the cage height, while SVR was selected for predicting PI-LL.

### 3.3. Final Model

#### 3.3.1. Feature Selection

**Figure 3:**
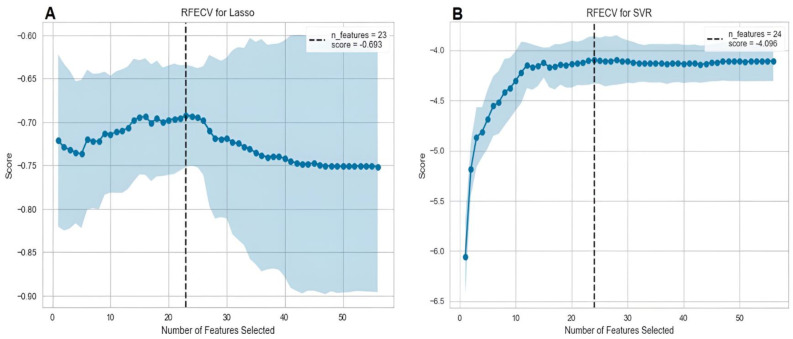
RFECV curves of two baseline models with negative MAEs for different numbers of features: (**A**) an LR model for interbody cage height prediction and (**B**) an SVR model for postoperative PI-LL prediction.

Figure 3 depicts the RFECV results for two baseline modes. In the LR model for predicting interbody cage height, the RFE curve identified 23 features as the optimal input for achieving peak performance, with a negative optimum cut-off MAE of −0.693. Likewise, the SVR model for predicting postoperative PI-LL identified 24 features as the optimal number, with a negative cut-off MAE of −4.096. The two subsets of features were subsequently employed to retrain the models (Supplementary Table S3), and the final models underwent validation using the testing set.

#### 3.3.2. Optimal Model Performance

**Figure 4:**
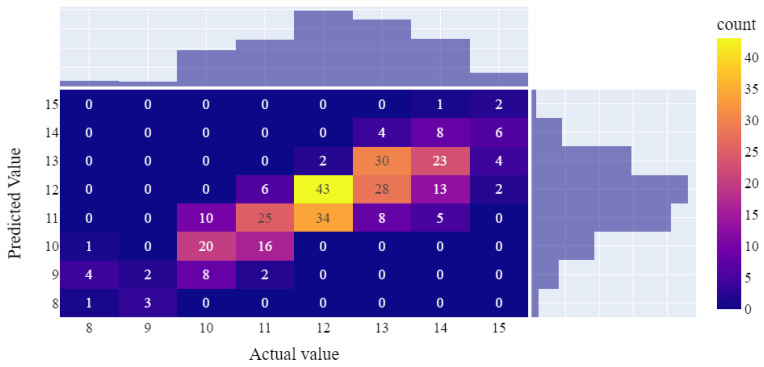
Confusion matrix for final model performance in the prediction of interbody cage height.

As shown in Table 3, the finalized LASSO algorithm for cage height prediction demonstrated an RMSE of 1.01 and an MAE of 0.7. These values reflect an enhancement over the metrics obtained prior to feature reduction (i.e., 1.06 and 0.76, respectively). Figure 4 depicts the accuracy of cage height prediction using the testing set, with 42.12% (131/311) of cases achieving exact values. Our model demonstrated commendable accuracy for interbody cage heights with the range of 10–13 mm. Notably, the most accurate prediction was obtained for a height of 12 mm, with 54.43% (43/79) of cases accurately predicted. Simultaneously, the accuracy ratios for sizes 10, 11, and 13 mm were 52.63% (20 of 38 cases), 51.02% (25 of 49 cases), and 42.86% (30 of 70 cases), respectively. In the majority of the remaining cases, the model exhibited a 1 mm prediction error, resulting in an overall accuracy rate of 88.75% (276 out of 311 cases) within the acceptable margin of 1 mm.

Due to the limited sample sizes in the 8, 9, and 15 mm fusion cage groups, the model encountered elevated prediction errors. Specifically, four of the six cases with an actual cage height of 8 mm were erroneously predicted to have a height of 9 mm. Within the 9 mm group, predicted values were 8 mm in three cases and 9 mm in two cases. Notably, for the 15 mm group, the model tended to predict interbody cage heights within the range of 13 to 14 mm in 10 out of 14 cases.

**Figure 5:**
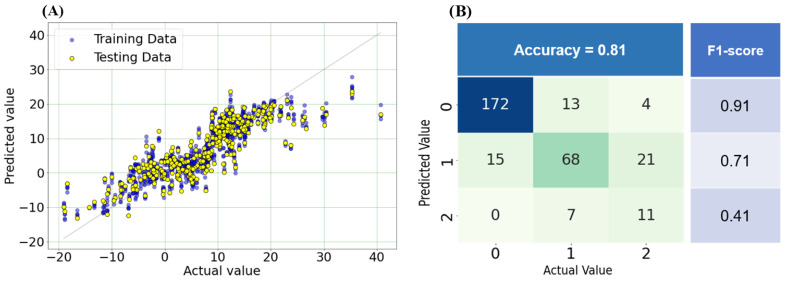
Performance of SVR. (**A**) Calibration plot (actual and predicted values) for predicting postoperative PI-LL on both training and testing data. (**B**) Confusion matrix for stratifying postoperative PI-LL on the testing set into three groups: 0 (<10), 1 (10–20), and 2 (>20).

Moving on to postoperative PI-LL prediction, the final SVR model achieved lower RMSE and MAE values on the testing set compared to the baseline model (5.19 and 3.86 versus 5.4 and 4.15; Table 3). In Figure 5A, the model’s performance on both the training and testing data is depicted, indicating a well-calibrated model where most points cluster around the regression line. This observation suggests close alignment between predicted PI-LL values and actual values. However, in cases with PI-LL values exceeding 20, more considerable errors were observed. Furthermore, the model exhibited high precision in stratifying postoperative PI-LL, achieving an accuracy of 0.81 and a high F1-score for the 0 group (Figure 5B).

#### 3.3.3. Feature Importance

**Figure 6:**
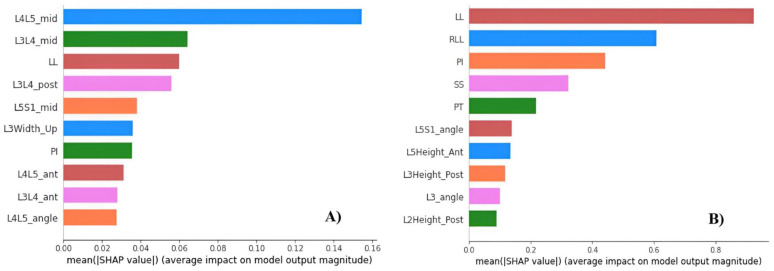
(**A**) Most crucial features for the model of interbody cage height prediction. (**B**) Most crucial features for the model of postoperative PI-LL prediction. Note: The explanations of feature abbreviations are provided in Supplementary Table S1.

Figure 6 visualizes the ten most influential features in the two final models. In predicting interbody cage height, the intervertebral height at the midpoint of L4–L5 (L4L5_mid) emerged as the most crucial factor. This prediction was notably influenced by three angles: LL, PI, and the L4–L5 intervertebral disc angle (L4L5_angle). Additionally, crucial parameters included the intervertebral heights of lumbar segments from L3 to S1, encompassing the intervertebral height at the midpoint of L3–L4 and L5-S1 (L3L4_mid and L5S1_mid), the posterior intervertebral height of L3–L4 (L3L4_post), and the anterior intervertebral height of L3–L4 and L4–L5 (L3L4_ant and L4L5_ant). Among the factors related to vertebral body size, only the upper vertebral width of L3 (L3Width_up) was included in this influential list.

Preoperative LL, relative LL (RLL), and PI played crucial roles in predicting postoperative PI-LL. Essential features associated with PI-LL after surgery predominantly involved angles related to preoperative sagittal alignment, such as sacrum slope (SS), pelvic tilt (PT), and L5-S1 intervertebral disc angle (L5S1_angle). Additionally, factors influencing PI-LL prediction were linked to the height of the vertebral body, including the anterior height of the L5 vertebra and the posterior height of the L2 and L3 vertebrae (L2Height_Post and L3Height_Post).

## 4. Discussion

Spinopelvic alignment restoration is essential for both adult spinal deformity surgery and short-segment lumbar interbody fusion [8,34,35]. However, determining the influence of each factor on sagittal alignment is difficult because the normal standing posture is jointly determined by multiple lumbosacral factors [36,37]. As shown in Figure 6 in the present study, the postoperative value of PI-LL is substantially influenced by the preoperative values of LL, RLL, and PI. However, because the PI value is regarded as a constant anatomic feature with slight variation in pathologic disorders or lumbar spine interventions [38], determining the postoperative LL is typically necessary for predicting the optimal PI-LL. According to previous research, LL restoration after surgery is closely linked to preoperative LL and PI [39,40,41,42]. Therefore, LL and PI can be used to predict the LL and PI-LL values after surgery, as in our model.

Appropriate parameters must be obtained for enhancing surgical quality, and surgeons must develop effective strategies to achieve harmonious sagittal alignment. Our model demonstrated a strong capacity to generate a satisfactory PI-LL value while being able to forecast the potential range of this value. By selecting patients without ASD for the dataset, the algorithm trained on these data was able to generate a favorable PI-LL value, which can be used to reduce the incidence of ASD in patients [7]. Our PI-LL prediction model was also able to provide predictions for surgical planning in selecting the appropriate surgical technique and instruments. Actually, the optimal PI-LL has been the subject of debate. Satoshi et al. [43] reported that this value is inconsistent. Meanwhile, multiple studies have suggested that surgeons must strive to reduce PI-LL to 10° or less whenever possible [8,44,45]. According to our model, if unsatisfactory PI-LL prediction values are obtained before surgery, surgeons could consider implementing additional intraoperative techniques. To achieve an adequate LL value, strong fixation with a curved rod system can be implemented. In some cases of severe hypolordosis, osteotomy techniques such as pedicle subtraction osteotomy are also a viable option [46]. Furthermore, the predictive results of postoperative PI-LL from our algorithm may aid in rod bending or in the determination of the number of spinal levels requiring fixation when a surgeon receives intraoperative fluoroscopic images. However, previous studies have revealed substantial discrepancies between standing and prone angle measurements [47,48]. Therefore, these models must be further developed to ensure their seamless integration from preoperative planning to actual surgery.

Size, shape, and position play a crucial role in the insertion of an intervertebral cage. However, findings regarding the importance of the implant shape and placement have been inconsistent. Cage lordosis and final LL after surgery are strongly correlated, with a more anterior placement resulting in greater intervertebral lordosis [18]. Conversely, some in vitro biomechanical and clinical studies have reported that the cage position and geometry do not affect sagittal alignment after lumbar interbody fusion [49,50,51]. The cage height typically serves as a key factor applied by surgeons for improving lordosis [52,53], and our research has primarily focused on predicting this index. Most of our cage height values were between 12 and 13 mm, which are consistent with the recommended cage heights of 11, 12, or 13 mm for the L3–L4 and L4–L5 levels in a previous study conducted in the Chinese population [16]. In addition, our model performed well for cases within this range, indicating its potential clinical applicability for the Asian population. Overall, predicting the appropriate interbody cage size can assist surgeons in decision-making and improve postoperative outcomes, particularly for inexperienced surgeons. Prediction using our model can also provide the cage height with an error of approximately 1 mm only (Figure 4). Consequently, fewer cages need to be sterilized, thus reducing the costs of surgery. In addition, the costs of treatment decrease due to the reduced operation duration and complication rates. Therefore, patients evidently benefit from the development of these models.

Our results indicated that the disc height of the pathological segment and the two adjacent levels plays a crucial role in predicting the height of the interbody cage (Figure 6). To predict this value, Wang et al. [16] developed a regression model that emphasizes the importance of the intervertebral height at the midpoint of the pathological segment (MIVH): interbody cage height = 11.123 − 0.563*gender + 0.149*MIVH. In our study, gender was one of the final 23 features used to build the optimal model, but its influence was not as evident as that of the other parameters. With the exception of the parameters associated with the intervertebral disc height, PI and LL contributed to the prediction of the interbody cage height. These two parameters also contributed to the aforementioned prediction of postoperative PI-LL. Lafage et al. [11] discovered that pelvic retroversion and global sagittal balance in adult patients with spinal deformities were primarily influenced by the PI and LL values. Here, we emphasized that PI and LL are among the most crucial parameters for both long- and short-segment fusion surgeries.

Multiple researchers have attempted to develop algorithms for predicting postoperative sagittal parameters and the interbody cage height, aiming to enhance accuracy and applicability in clinical practice. Traditionally, these formulas featured a limited set of variables to simplify computations. Lafage et al. [11,54] developed one of the most accurate formulas for predicting the sagittal vertical axis (SVA). They used only four variables in their formula: PI, LL, thoracic kyphosis, and age. Legaye and Duval-Beaupère [38,55] proposed multilinear regression models for calculating LL by using only basic parameters, such as thoracic kyphosis, SS, PI, PT, and T9 spinopelvic inclination. In contrast to prior approaches, our goal was to incorporate all significant lumbar parameters into algorithm development. Because our prediction models (LR for the interbody cage height and SVR for postoperative PI-LL) and previous models share the same characteristic of utilizing multiple linear algorithms, we took advantage of the current technological advancements to incorporate as many variables as possible. However, certain factors, such as the width and length of the vertebral body, were found to be crucial features in our model, a novel finding absent from the existing medical literature. While this discovery might be serendipitous during model training with our dataset, it necessitates further verification in subsequent research. Previously, employing multiple parameters may have been impractical for routine clinical use. However, leveraging computational power, contemporary methods now facilitate improved predictive accuracy, rendering these predictions applicable in clinical scenarios. According to Langella et al. [56], computer-assisted methods are associated with a failure rate below 20% for predicting PI and SVA. To the best of our knowledge, this study is pioneering in presenting a pipeline and diverse models for predicting PI-LL and cage height from preoperative X-ray images through AI.

This study has several limitations. Firstly, it is imperative to acknowledge the retrospective nature and single-center design, which inherently presents constraints due to a modest sample size. As a result, optimal interbody cage height or postoperative PI-LL may be subject to variability influenced by subjective factors such as the surgeon’s technique and patient demographics. Moreover, the small sample size and unbalanced data posed challenges in achieving satisfactory accuracy for certain cases despite employing various resampling techniques. Although these methods were applied, they did not yield improved results, highlighting the necessity to expand the sample size and explore alternative strategies for addressing imbalanced data. Despite these constraints, the introduction of multiple algorithms in this study introduced a pioneering concept, setting the groundwork for enhanced predictive accuracy in future multicenter studies. Moreover, subsequent research should aim to validate our model in diverse patient populations to ensure its reliability across different patient profiles. Secondly, this study was limited to patients with monosegmental TLIF at the L4–L5 level, and only one sagittal parameter, PI-LL, was predicted. However, using our algorithms, a large number of postoperative parameters can be predicted not only for single-level fusion surgery but also for surgeries involving multiple levels. Thirdly, sagittal balance is associated with factors such as SVA, T1 spinopelvic inclination, and C7 plumb line, which are evaluated using full-length spine radiographs [45,57,58]. Because we focused only on short-segment fusion, we examined only the lumbar region. Therefore, global sagittal balance factors must be examined for TLIF surgery in the future. Lastly, the complexity of our model, involving multiple steps, increases the probability of errors. Additionally, the accuracy of features extracted from X-rays through AI could be enhanced by incorporating successful models for vertebral body segmentation from MRI or CT images, as demonstrated in recent studies [59,60].To increase predictive accuracy, a synthetic model must be developed, integrating radiographic parameters from X-ray, MRI, and CT scans. This comprehensive approach will contribute to refining and validating the predictive capabilities of our model in diverse clinical scenarios.

## 5. Conclusions

This study marks a significant stride in the development of end-to-end AI models tailored for predicting interbody cage height and postoperative PI-LL in TLIF surgery. Our findings underscore the efficacy of sophisticated computer-assisted models in spinal morphometry, showcasing the remarkable accuracy of ML algorithms. These models emerge as valuable tools for surgeons, offering substantial support in both preoperative planning and postoperative assessment. Our results highlight the significance of integrating multiple crucial parameters, particularly preoperative PI and LL, into multilinear regression equations. This innovative approach demonstrates promise in predicting outcomes for spinal fusion surgery, emphasizing the potential for improved precision in patient-specific treatment strategies. However, to ensure model reliability and generalizability, further validation and refinement with larger datasets and multicenter studies are required.

## Figures and Tables

**Table 1:** Hyperparameter optimization for ML algorithms for the prediction of interbody cage height and postoperative PI-LL.

ML Algorithm	Hyperparameter Ranges	Optimal Values for Cage Height Prediction	Optimal Values for PI-LL Prediction
LR	Alpha = [0, 1], interval = 0.001	0.001	0.01
DT	Criterion = [squared_error, friedman_mse, absolute_error, poisson] min_samples_split = [10, 20, 30, 40, 50] min_samples_leaf = [5, 10, 20, 30, 40]	poisson 30 20	squared_error 50 5
SVR	kernels = [poly, linear, rbf, sigmoid] C = [0.1, 1, 10, 100] gamma = [0.001, 0.01, 0.1, 1]	sigmoid 10 0.001	linear 0.1 1
MLP	hidden_layer_sizes = [(50, 50, 50), (100, 100, 100), (200, 200, 200)] activation = [tanh, relu] solver = [sgd, adam, lbfgs] alpha = [0.0001, 0.001, 0.05]	(200, 200, 200) relu lbfgs 0.05	(200, 200, 200) tanh sgd 0.0001
KNN	n_neighbors = [5, 10, 20, 30, 40, 50] metric = [euclidean, manhattan, minkowski] weights = [uniform, distance]	20 euclidean uniform	5 euclidean distance

**Table 2:** Performance of ML algorithms in the prediction of interbody cage height and postoperative PI-LL. RMSE: root mean square error; MAE: mean absolute error.

Algorithm	Cage Height		Postoperative PI-LL	
	RMSE	MAE	RMSE	MAE
DT	1.11 ± 0.042	0.85 ± 0.038	7.05 ± 0.85	5.39 ± 0.72
LR	1.06 ± 0.011	0.76 ± 0.01	5.42 ± 0.56	4.2 ± 0.42
SVR	1.09 ± 0.008	0.77 ± 0.01	5.4 ± 0.52	4.15 ± 0.48
MLP	1.16 ± 0.02	0.87 ± 0.016	6.36 ± 0.8	4.84 ± 0.76
KNN	1.11 ± 0.042	0.85 ± 0.038	7.05 ± 0.85	5.39 ± 0.72

**Table 3:** Performance of two final models. RMSE: root mean square error; MAE: mean absolute error.

	Baseline Model Performance		Optimal Model Performance	
	RMSE	MAE	RMSE	MAE
Cage height prediction	1.06	0.76	1.01	0.7
Postoperative PI-LL prediction	5.4	4.15	5.19	3.86

## Data Availability

Access to the dataset shall be provided by the corresponding authors upon reasonable request and in accordance with the policies of the relevant institution.

## Supplementary Materials

The following supporting information can be downloaded at https://www.mdpi.com/article/10.3390/bioengineering11020164/s1; Table S1: Spinal parameter features extracted using a deep learning model; Table S2: ICCs validating the reliability of the deep learning model in measuring bone distance parameters compared with the MRI results; Table S3: Two subsets of crucial features for two baseline ML models.
