# Supplementary material for: Development of End-to-End Artificial Intelligence Models for Surgical Planning in Transforaminal Lumbar Interbody Fusion
Source: Bioengineering (Basel). 2024 Feb 8;11(2):164. doi: 10.3390/bioengineering11020164 (PMC10885900; doi:10.3390/bioengineering11020164)
Supplement: Supplementary file 1 [file bioengineering-11-00164-s001.zip › bioengineering-2810760-supplementary.pdf]

Table S1. Spinal parameter features extracted using a deep learning model.

No.	Feature	Abbreviation	Description
1	Anterior intervertebral height at the L1-L2 level	L1L2_ant	Distance of the perpendicular line extending from the anterior edge of the L1 inferior endplate to the straight line tangent to the L2 superior endplate
2	Intervertebral height at the midpoint of the L1-L2 level	L1L2_mid	Distance of the perpendicular line extending from the midpoint of the L1 inferior endplate to the straight line tangent to the L2 superior endplate
3	Posterior intervertebral height at the L1-L2 level	L1L2_post	Distance of the perpendicular line extending from the posterior edge of the L1 inferior endplate to the straight line tangent to the L2 superior endplate
4	Anterior intervertebral height at the L2-L3 level	L2L3_ant	Distance of the perpendicular line extending from the anterior edge of the L2 inferior endplate to the straight line tangent to the L3 superior endplate
5	Intervertebral height at the midpoint of the L2-L3 level	L2L3_mid	Distance of the perpendicular line extending from the midpoint of the L2 inferior endplate to the straight line tangent to the L3 superior endplate
6	Posterior intervertebral height at the L2-L3 level	L2L3_post	Distance of the perpendicular line extending from the posterior edge of the L2 inferior endplate to the straight line tangent to the L3 superior endplate
7	Anterior intervertebral height at the L3-L4 level	L3L4_ant	Distance of the perpendicular line extending from the anterior edge of the L3 inferior endplate to the straight line tangent to the L4 superior endplate
8	Intervertebral height at the midpoint of the L3-L4 level	L3L4_mid	Distance of the perpendicular line extending from the midpoint of the L3 inferior endplate to the straight line tangent to the L4 superior endplate
9	Posterior intervertebral height at the L3-L4 level	L3L4_post	Distance of the perpendicular line extending from the posterior edge of the L3 inferior endplate to the straight line tangent to the L4 superior endplate
10	Anterior intervertebral height at the L4-L5 level	L4L5_ant	Distance of the perpendicular line extending from the anterior edge of the L4 inferior endplate to the straight line tangent to the L5 superior endplate
11	Intervertebral height at the midpoint of the L4-L5 level	L4L5_mid	Distance of the perpendicular line extending from the midpoint of the L4 inferior endplate to the straight line tangent to the L5 superior endplate
12	Posterior intervertebral height at the L4-L5 level	L4L5_post	Distance of the perpendicular line extending from the posterior edge of the L4 inferior endplate to the straight line tangent to the L5 superior endplate
13	Anterior intervertebral height at the L5-S1 level	L5S1_ant	Distance of the perpendicular line extending from the anterior edge of the L5 inferior endplate to the straight line tangent to the S1 superior endplate
14	Intervertebral height at the midpoint of the L5-S1 level	L5S1_mid	Distance of the perpendicular line extending from the midpoint of the L5 inferior endplate to the straight line tangent to the S1 superior endplate
15	Posterior intervertebral height at the L5-S1 level	L5S1_post	Distance of the perpendicular line extending from the posterior edge of the L5 inferior endplate to the straight line tangent to the S1 superior endplate
16	Pelvic incidence	PI	Angle between the line drawn perpendicularly to the sacral endplate at its midpoint and the line joining the center of the femoral heads (midpoint of the bicoxofemoral axis) to the same midpoint of the sacral endplate
17	Lumbar lordosis	LL	Angle between the superior endplates of L1 and S1
18	Pelvic incidence minus lumbar lordosis before surgery	PImLL_before	Subtraction of preoperative pelvic incidence and preoperative lumbar lordosis

19	Lower lumbar lordotic arc	L4S1_angle	Angle between the superior endplates of L4 and S1
20	Lordosis distribution index	LDI	Magnitude of lower-arc lordosis relative to total lordosis, calculated using the formula L4-S1 lordosis/L1-S1 lordosis × 100%
21	Relative lumbar lordosis	RLL	Subtraction of measured LL and optimal LL (optimal LL = PI × 0.62 + 29)
22	Sacral slope	SS	Angle between the sacral endplate and the horizontal
23	Pelvic tilt	PT	Angle between the line joining the midpoint of the sacral endplate to the midpoint of the bicoxofemoral axis and the vertical
24	L1 vertebral angle	L1_angle	Angle between the superior and inferior endplates of L1
25	L2 vertebral angle	L2_angle	Angle between the superior and inferior endplates of L2
26	L3 vertebral angle	L3_angle	Angle between the superior and inferior endplates of L3
27	L4 vertebral angle	L4_angle	Angle between the superior and inferior endplates of L4
28	L5 vertebral angle	L5_angle	Angle between the superior and inferior endplates of L5
29	L1-L2 intervertebral disc angle	L1L2_angle	Angle between the inferior endplate of L1 and the superior endplate of L2
30	L2-L3 intervertebral disc angle	L2L3_angle	Angle between the inferior endplate of L2 and the superior endplate of L3
31	L3-L4 intervertebral disc angle	L3L4_angle	Angle between the inferior endplate of L3 and the superior endplate of L4
32	L4-L5 intervertebral disc angle	L4L5_angle	Angle between the inferior endplate of L4 and the superior endplate of L5
33	L5-S1 intervertebral disc angle	L5S1_angle	Angle between the inferior endplate of L5 and the superior endplate of S1
34	Upper vertebral width of L1	L1Width_Up	Distance from the anterior to the posterior edge of the L1 superior endplate
35	Lower vertebral width of L1	L1Width_Down	Distance from the anterior to the posterior edge of the L1 inferior endplate
36	Upper vertebral width of L2	L2Width_Up	Distance from the anterior to the posterior edge of the L2 superior endplate
37	Lower vertebral width of L2	L2Width_Down	Distance from the anterior to the posterior edge of the L2 inferior endplate
38	Upper vertebral width of L3	L3Width_Up	Distance from the anterior to the posterior edge of the L3 superior endplate
39	Lower vertebral width of L3	L3Width_Down	Distance from the anterior to the posterior edge of the L3 inferior endplate
40	Upper vertebral width of L4	L4Width_Up	Distance from the anterior to the posterior edge of the L4 superior endplate
41	Lower vertebral width of L4	L4Width_Down	Distance from the anterior to the posterior edge of the L4 inferior endplate
42	Upper vertebral width of L5	L5Width_Up	Distance from the anterior to the posterior edge of the L5 superior endplate
43	Lower vertebral width of L5	L5Width_Down	Distance from the anterior to the posterior edge of the L5 inferior endplate
44	Anterior height of L1	L1Height_Ant	Distance from the anterior edge of the L1 superior endplate to the anterior edge of the L1 inferior endplate
45	Posterior height of L1	L1Height_Post	Distance from the posterior edge of the L1 superior endplate to the posterior edge of the L1 inferior endplate
46	Anterior height of L2	L2Height_Ant	Distance from the anterior edge of the L2 superior endplate to the anterior edge of the L2 inferior endplate
47	Posterior height of L2	L2Height_Post	Distance from the posterior edge of the L2 superior endplate to the posterior edge of the L2 inferior endplate
48	Anterior height of L3	L3Height_Ant	Distance from the anterior edge of the L3 superior endplate to the anterior edge of the L3 inferior endplate
49	Posterior height of L3	L3Height_Post	Distance from the posterior edge of the L3 superior endplate to the posterior edge of the L3 inferior endplate
50	Anterior height of L4	L4Height_Ant	Distance from the anterior edge of the L4 superior endplate to the anterior edge of the L4 inferior endplate
51	Posterior height of L4	L4Height_Post	Distance from the posterior edge of the L4 superior endplate to the posterior edge of the L4 inferior endplate
52	Anterior height of L5	L5Height_Ant	Distance from the anterior edge of the L5 superior endplate to the anterior edge of the L5 inferior endplate
53	Posterior height of L5	L5Height_Post	Distance from the posterior edge of the L5 superior endplate to the posterior edge of the L5 inferior endplate

Table S2. ICCs validating the reliability of the deep learning model in measuring bone distance parameters compared with the MRI results of two authors (Anh Tuan Bui, Giam Minh Trinh).

No.	Feature	ICC (95% confidence interval)
1	L1Width_Up	0.929 (0.913–0.942)
2	L1Width_Down	0.941 (0.928–0.952)
3	L2Width_Up	0.879 (0.653–0.941)
4	L2Width_Down	0.829 (0.763–0.874)
5	L3Width_Up	0.905 (0.883–0.923)
6	L3Width_Down	0.914 (0.895–0.93)
7	L4Width_Up	0.89 (0.867–0.91)
8	L4Width_Down	0.901 (0.881–0.919)
9	L5Width_Up	0.86 (0.831–0.886)
10	L5Width_Down	0.855 (0.823–0.881)
11	L1Height_Ant	0.831 (0.794–0.862)
12	L1Height_Post	0.887 (0.863–0.908)
13	L2Height_Ant	0.881 (0.851–0.905)
14	L2Height_Post	0.78 (0.723–0.824)
15	L3Height_Ant	0.79 (0.738–0.831)
16	L3Height_Post	0.947 (0.936–0.957)
17	L4Height_Ant	0.921 (0.904–0.936)
18	L4Height_Post	0.84 (0.807–0.869)
19	L5Height_Ant	0.945 (0.934–0.955)
20	L5Height_Post	0.832 (0.772–0.874)
21	L1L2_ant	0.925 (0.877–0.951)
22	L1L2_mid	0.883 (0.857–0.905)
23	L1L2_post	0.881 (0.856–0.903)
24	L2L3_ant	0.916 (0.898–0.931)
25	L2L3_mid	0.897 (0.876–0.916)
26	L2L3_post	0.915 (0.898–0.93)
27	L3L4_ant	0.905 (0.884–0.923)
28	L3L4_mid	0.885 (0.859–0.906)
29	L3L4_post	0.935 (0.92–0.948)
30	L4L5_ant	0.945 (0.92–0.96)
31	L4L5_mid	0.908 (0.877–0.931)
32	L4L5_post	0.909 (0.89–0.925)
33	L5S1_ant	0.917 (0.899–0.932)
34	L5S1_mid	0.929 (0.914–0.942)
35	L5S1_post	0.921 (0.904–0.935)

Table S3. Two subsets of crucial features for two baseline ML models.

No.	Cage height prediction model	Postoperative PI-LL prediction model
1	L4L5_mid	LL
2	L3L4_mid	RLL
3	LL	PI
4	L3L4_post	SS
5	L5S1_mid	PT
6	L3Width_Up	L5S1_angle
7	PI	L5Height_Ant
8	L4L5_ant	L3Height_Post
9	L3L4_ant	L3_angle
10	L4L5_angle	L2Height_Post
11	PImLL_before	Age
12	Gender	L4S1_angle

13	L2L3_angle	L1L2_angle
14	LDI	L3L4_angle
15	L3_angle	PImLL_before
16	L4S1_angle	L4L5_angle
17	L2Height_Ant	L1Width_Up
18	L3Width_Down	L3Width_Down
19	L2Height_Post	L4Width_Up
20	L5_angle	L4Height_Ant
21	L4Width_Down	L1_angle
22	L5Width_Down	L4L5_post
23	L2L3_post	Gender
24	—	Body Height
